# Cortical processing of object affordances for self and others' action

**DOI:** 10.3389/fpsyg.2014.00538

**Published:** 2014-06-17

**Authors:** Monica Maranesi, Luca Bonini, Leonardo Fogassi

**Affiliations:** ^1^Brain Center for Social and Motor Cognition, Italian Institute of TechnologyParma, Italy; ^2^Department of Neuroscience, University of ParmaParma, Italy

**Keywords:** perception, space, sensorimotor transformation, visual streams, grasping

## Abstract

The perception of objects does not rely only on visual brain areas, but also involves cortical motor regions. In particular, different parietal and premotor areas host neurons discharging during both object observation and grasping. Most of these cells often show similar visual and motor selectivity for a specific object (or set of objects), suggesting that they might play a crucial role in representing the “potential motor act” afforded by the object. The existence of such a mechanism for the visuomotor transformation of object physical properties in the most appropriate motor plan for interacting with them has been convincingly demonstrated in humans as well. Interestingly, human studies have shown that visually presented objects can automatically trigger the representation of an action provided that they are located within the observer's reaching space (peripersonal space). The “affordance effect” also occurs when the presented object is outside the observer's peripersonal space, but inside the peripersonal space of an observed agent. These findings recently received direct support by single neuron studies in monkey, indicating that space-constrained processing of objects in the ventral premotor cortex might be relevant to represent objects as potential targets for one's own or others' action.

## Introduction

Perception and action have been considered for a long time as two serially organized steps of processing, with the former relying on sensory brain areas and the latter implemented by the motor cortex. In this view, cognition would emerge as an intermediate step of information processing performed by associative cortical areas. This classical “sandwich model” (Hurley, 1998), in which perception and action do never directly interact one with the other, has been challenged by a growing body of evidence in the last three decades (see Goodale and Milner, 1992; Rizzolatti and Matelli, 2003). These studies suggest that a crucial role in perception is played by cortical motor regions as well, especially when sensory information is required for acting. An intriguing synthesis of this view maintains that “perception is not something that happens to us, or in us: It is something we do” (Noë, 2004).

The tight link of perceptual processes with the motor ones has a particularly elegant exemplification in the concept of “affordance”, coined by the psychologist James Gibson (1979). According to Gibson, affordances are all the motor possibilities that an object in the environment offers an individual: crucially, they depend on the motor capabilities of the observer but not on his/her intentions or needs. Among the different possible affordances of an object, the one that will prevail and will be more likely turned into an overtly executed action depends upon the contextual situation, the goals and intentions of the perceiver. For example, a cup might afford grasping of its handle or of its body if one expects it contains a hot or cold drink, respectively. In addition, it might also afford grasping of its top, if it is empty and the agent wants simply to move it away. In all these cases, two types of parallel processing of the object take place: its semantic description, provided by higher order cortical visual areas, and a pragmatic description, which includes the extraction of its various affordances and micro-affordances (Ellis and Tucker, 2000), and their possible translation into action (Jeannerod et al., 1995).

Which are the cortical regions involved in the processing of objects affordances? Goodale and Milner (1992) modified the Ungerleider and Mishkin's proposal of the two visual streams (1982), suggesting that the “ventral stream”, linking primary visual cortex to the inferotemporal regions, is responsible for object recognition, while the “dorsal stream”, ending in the posterior parietal region, plays a crucial role in the sensorimotor transformations for visually guided object-directed actions. Based on clinical, functional and anatomical data, Rizzolatti and Matelli (2003) proposed to further subdivide the dorsal stream into two distinct functional systems, formed by partially segregated cortical pathways: the dorso-dorsal (d-d) and the ventro-dorsal (v-d) stream. According to their proposal, the d-d stream would correspond to the dorsal stream as previously defined by Milner and Goodale, exploiting sensory information for the control of reaching movements in space, while the v-d stream would be specifically involved in sensorimotor transformation for grasping, space perception and action recognition. Thus, also within the originally defined dorsal stream, there is a subsystem, the v-d stream, which might play a role in perceptual functions.

## From object affordances to sensorimotor transformations: parallel parieto-frontal circuits

Object grasping is one of the most frequently performed and highly specialized behavior in primates (Jeannerod et al., 1995; Macfarlane and Graziano, 2009). One of the most challenging aspects in the control of grasping is the configuration of the hand according to the object features during the reaching phase (Jeannerod et al., 1995). Jeannerod (1984) and Arbib (1985), independently, proposed the existence of two specific neural systems responsible for the reaching and grasping components of reach-to-grasp actions. In the last decades, several studies on both humans and monkeys have been carried out in order to identify and describe the cortical mechanisms underlying such a complex sensorimotor transformation. While most of these studies aimed at clarifying the role of areas of the v-d stream, particularly of the anterior intraparietal area (AIP) and ventral premotor area F5, recent findings shed new light on the possible involvement of areas belonging to the dorso-dorsal stream (parietal area V6A and dorsal premotor area F2) in the visuomotor transformations involved in grasping actions.

### The AIP-F5 circuit

From the early ‘90s, Sakata and colleagues have investigated monkey parietal cortex by means of a paradigm designed to study neuronal activity while the monkey had to observe and subsequently grasp objects of different size and shape (Taira et al., 1990; Sakata et al., 1995; Murata et al., 2000). This condition could be performed in the light or in the dark, in separate sessions. Moreover, the task also included a condition in which the monkey had to simply fixate the object, without performing any grasping movement. The authors were able to describe, as in their previous studies, two types of visually-modulated neurons: “visual-dominant” neurons, which discharged during grasping in the light but not in the dark, and “visual-motor” neurons, which fired also during grasping in the dark, although weaker compared with the same action performed in the light. Within both these two populations of neurons, they further subdivided neurons in “object-type” or “non-object-type,” depending on whether or not they responded to object presentation during the fixation task. Interestingly, the discharge of many object-type neurons exhibited the same preference for a given object (or set of objects) during both object fixation and grasping. This finding suggests that object-type neurons play a crucial role in the visuomotor transformation of object affordances in the most appropriate hand shape for grasping. Their response and the preserved object selectivity, also during trials in which the monkey did not perform any action, further indicate that the neural mechanisms for the extraction of object affordances rely on the monkey motor possibilities, but not necessarily on its actual execution of a grasping action. Therefore, also the dorsal pathway (in particular the ventro-dorsal stream), appears to play a role in object perception.

Another study demonstrated a causal role of area AIP in computing object properties for adjusting the finger posture according to the size and shape of the target object (Gallese et al., 1994). In this study, muscimol (a GABA-agonist which inhibits neurons activity) was injected in monkey area AIP, showing that while the arm reaching component was unimpaired, the hand shaping for grasping objects, particularly the small ones, was clearly altered, and associated with a reduced movement speed. The affected grip could be subsequently corrected by the monkey based on tactile exploration of the target object, suggesting that the deficit specifically concerns the visuomotor transformation for hand grasping.

What is the anatomo-functional mechanism through which the perceptual description of an object accesses the motor representations necessary for turning it into the most appropriate hand shape? Anatomical studies based on tracers injections in AIP have shown that this area is linked to many others through monosynaptic connections. In particular, they showed that area AIP forms an anatomo-functional module with the ventral premotor area F5 (Luppino et al., 1999; Borra et al., 2008).

Neurophysiological studies showed that area F5 contains neurons discharging during specific goal-related motor acts (Rizzolatti et al., 1988). Moreover, similarly to area AIP, F5 visuomotor neurons discharge to the visual presentation of graspable objects, often with a clear selectivity for their size and shape (Murata et al., 1997; Raos et al., 2006). These neurons have been defined as “canonical” neurons (Rizzolatti and Fadiga, 1998). Interestingly, both during object fixation and grasping in the dark, F5 neurons maintained the same selectivity for a given object or set of objects (Raos et al., 2006), reflecting a visuomotor matching mechanism as the one previously described for area AIP. In contrast to area AIP, however, no F5 neurons were recorded discharging only during grasping in the light and also to object presentation. In addition, while AIP visual responses to objects appear to encode the geometrical features shared by the different objects (Murata et al., 2000), F5 visual responses reflect the parameters of hand configuration shared by different types of grip (Raos et al., 2006). In line with these findings, muscimol inactivation of the F5 sector buried in the bank of the arcuate sulcus (F5p—Belmalih et al., 2009), which is more tightly linked with area AIP than F5 convexity (Luppino et al., 1999; Borra et al., 2008), produced a markedly impaired shaping of the hand during grasping (Fogassi et al., 2001). In particular, monkey were unable to produce the fingers configuration appropriate for the size and shape of the to-be-grasped object and, similarly to what previously described following inactivation of area AIP, the monkey could accomplish object grasping only by means of tactile feedback obtained through hand-object exploration.

Human studies revealed the existence of a putative homolog of monkey's area AIP in the anterior portion of the intraparietal sulcus (aIPS—Culham et al., 2003; Frey et al., 2005), which becomes specifically active during visually guided grasping. Interestingly, studies using TMS applied to aIPS reported a disruption of goal-dependent kinematics during reach-to-grasp trials (Tunik et al., 2005). In particular, this study reported that, depending on which parameter had to be controlled in the ongoing trial (object size or orientation), TMS pulse delivered to aIPS specifically disrupted the online control of the correspondent parameters of hand kinematics. Importantly, this effect was selectively produced by stimulation of aIPS and not of other parietal regions. The anatomo-functional connectivity between AIP and ventral premotor (PMv, considered the human homolog of area F5) has been demonstrated also in humans by a TMS study (Davare et al., 2010). These authors induced an AIP virtual lesion by means of repetitive TMS. At the same time, they studied with another (paired-pulse) combined TMS technique the possible facilitation exerted by the ventral premotor (PMv) on the primary motor (M1) cortex. The results clearly indicated that PMv-M1 interactions during grasping are driven by information about object properties provided by AIP, demonstrating the existence of a causal transfer of information on object features between the human parietal (AIP) and the premotor (PMv) nodes of the visuomotor transformation network.

### Area V6A-F2 circuit

The parieto-frontal circuit formed by area V6A (Galletti et al., 1999; Fattori et al., 2001, 2005), and dorsal premotor area F2vr (Raos et al., 2003) constitutes a subdivision of the dorsal visual pathway (Galletti et al., 2003), deemed to play a role in the encoding of the arm direction toward different locations in space. Surprisingly, recent studies have demonstrated that the neural code of this circuit is not limited to reaching movements.

Indeed, area V6A also contains neurons modulated by wrist orientation (Fattori et al., 2009) and by hand shape (Fattori et al., 2010) during object grasping. In addition, single V6A neurons have been described responding also to the visual presentation of real objects (Fattori et al., 2012). In this latter study, the authors tested single neurons responses to object presentation within two different task contexts, similar to those previously employed to test AIP and F5 visuomotor neurons, namely: a passive “object viewing task,” in which the monkey had to passively fixate the visually presented object, and a “reach-to-grasp task,” in which object presentation was followed by object grasping. Results showed that 60% of area V6A neurons discharged to the presentation of objects, regardless of the task context. In addition, about half of them showed a preferential discharge for a particular object or set of objects. Although AIP and V6A neurons appear to be similar in this respect, two important differences emerged from this comparison. First, a greater number of AIP than V6A neurons showed object selectivity (45 vs. 25%, respectively). Second, while AIP visual responses encoded the geometric features shared by the observed objects, both during passive fixation and grasping tasks (Murata et al., 2000), object coding by V6A neurons showed an interesting interaction with the task context: in the object viewing task, V6A neurons encoded objects geometric features, like those of AIP, while during the reach-to-grasp task V6A neurons' responses reflected the features of the grip used for grasping a certain set of object, regardless of their geometric similarity.

Further studies revealed that neuronal activity in area V6A can also specify object position with high specificity for the peripersonal (reachable) space not only during reaching tasks (Fattori et al., 2001, 2005; Hadjidimitrakis et al., 2013), but also during passive fixation tasks (Hadjidimitrakis et al., 2011). In particular, Hadjidimitrakis et al. (2013) investigated object position coding according to different reference frames. In this study, the monkey had to reach a spot of light located at different distances and lateralities from the body, with its hand starting at two different initial positions (near to or far from the body). Results showed that the majority of V6A neurons encoded reach targets mainly based on a body-centered frame of reference or combined with information relative to the hand position.

Taken together, these findings suggest that both object features and its spatial position are encoded by V6A neurons, very likely playing a role in turning perceptual representations of geometrical and spatial properties of objects into the appropriate motor plans for interacting with them. In this respect, V6A contribution appears to be quite similar to that of area AIP. However, differently from AIP, area V6A has no direct anatomical connections with areas of the ventral visual stream (Gamberini et al., 2009; Passarelli et al., 2011), suggesting that it might play a more relevant role in monitoring the ongoing visuomotor transformations during reaching-grasping movements. The rapid recovery from reaching and grasping deficits produced by V6A bilateral lesions (Battaglini et al., 2002) is in line with this view. Area V6A is also strongly connected with the dorsal premotor area F2 (Matelli et al., 1998), thus forming a parieto-frontal circuit similar to the AIP-F5 one. Area F2 has been shown to play a role in the encoding of object features (Raos et al., 2004), as well as in specifying object location relative to the monkey's peri- or extrapersonal space (Fogassi et al., 1999). In particular, Raos et al. (2004) have investigated the possible role of neurons in the ventral part of area F2 (F2vr) in encoding object within the peripersonal (reaching) space by employing the same paradigm previously used to test F5 visuomotor neurons. Interestingly, the results evidenced that several visually responsive F2vr visuomotor neurons displayed object-selective visual responses congruent with their selectivity shown during reaching-grasping execution. The presence of slightly similar visuomotor properties in areas V6A and AIP, on one side, and F2vr and F5, on the other, is in line with the evidence that these pairs of areas have some reciprocal anatomical connections (Borra et al., 2008; Gamberini et al., 2009; Gerbella et al., 2011), indicating that the ventral and dorsal aspects of the dorsal stream are not completely segregated. Indeed, these findings support the idea that the V6A-F2vr circuit can process both object intrinsic (shape and size) and extrinsic (spatial location) features, thus extending to areas belonging to the dorsal visual stream (Galletti et al., 2003; Rizzolatti and Matelli, 2003) the functions of encoding object features and of monitoring object-directed actions.

Although the homology between monkey and human posterior parietal areas remains not completely clear (Silver and Kastner, 2009), recent indirect evidence suggest that object features, as well as their location in space, might be processed along the dorsal pathway not only for motor purposes. For example, Gallivan et al. (2009) showed that a reach-related area in the superior parieto-occipital cortex in human was more activated for objects located in the peripersonal space, even when passively observed. Another study evidenced that posterior parietal cortex activated during visual processing of objects not only when no action planning was involved, but even when the subjects' attention was drawn away from the stimuli (Konen and Kastner, 2008). In the same study, the top stages of both ventral and dorsal streams showed considerable invariance of their activation in relation to changes in stimulus features such as size and viewpoint, which generally affects the lower stages of both streams. More interestingly, activations in both the ventral and the dorsal stream during the presentation of three-dimensional shapes have been reported with fMRI even in anesthetized monkeys (Sereno et al., 2002). Together with an increasing number of studies (Xu and Chun, 2009; Zachariou et al., 2014) on cortical object processing, these findings suggest that object information in the dorsal pathways is not only processed with the purpose of guiding or monitoring sensorimotor transformations, but can also play some role in perceptual and cognitive functions.

## Visuomotor transformation or sensorimotor association? pragmatic and perceptual functions in object processing

What happens exactly in the brain when we observe a graspable object? One possibility is that, as described above, a graspable object is represented pictorially in visual brain areas and, simultaneously, its pragmatic description (visuomotor transformation) is activated in areas of the v-d stream. Alternatively, neurons discharging at the sight of a real object might simply reveal that a visuomotor association did occur, likely irrespective of the specific physical properties of the object itself. Based on this latter view, one would predict that both seeing the real object and an arbitrary cue signal (e.g., a colored spot of light) previously associated to a specific grip posture, might evoke the same visuomotor response.

A recent study provides interesting data that directly address this issue. Baumann et al. (2009) recorded single neurons in area AIP of monkeys performing a delayed grasping task. During this task, monkeys were presented with a handle (target object) in different orientations, and a colored LED (cue signal), which instructed the animal to subsequently perform a power or a precision grip. Results showed that AIP neurons could represent both the handle orientation and the instructed grip type immediately after the presentation of the visual stimulus, indicating that AIP neurons can process object features in a context-dependent fashion. A modified version of the task (cue separation task) enabled to study neuronal responses also when information on object orientation and the required grip type were separately presented. In particular, when the target object was presented first, visuomotor neurons became active regardless of the preference for power or precision grip that they exhibited in the delayed grasping task. In contrast, when the cue was presented first (and the object was not yet visible), this information was only weakly represented in area AIP, while it was strongly encoded thereafter, when the target object was revealed. Together with the data reviewed above (Sakata et al., 1995; Murata et al., 1996), these findings indicate that, besides transforming object properties into the appropriate grip type, AIP visuomotor neurons can also encode abstract information provided by any visual stimulus previously associated with a specific grip. However, both object- and context-driven transformations of visual information into an appropriate motor representation of a hand grip require that the object to be grasped be visible in front of the monkey. Thus, area AIP does not simply associate contextual visual stimuli with motor representations, but plays an active role in the processing of a pragmatic description of observed objects. Interestingly, even human fMRI studies showed that area AIP can activate during both the recognition and construction of three-dimensional shapes in the absence of visual guidance, but not during mental imagery of the same processes (Jancke et al., 2001), where overt sensory input and motor output are absent: this finding clearly supports the idea that the physical presence of the object is crucial for triggering area AIP neurons activity.

Do parallel processings of pictorial and pragmatic description of object features integrate or remain independent? Anatomical studies have demonstrated a rich pattern of connections linking temporal visual areas with inferior parietal regions belonging to the v-d stream (Borra et al., 2008, 2010). In addition, neurophysiological data on monkeys have revealed that a crucial aspect for both pictorial and pragmatic description of real objects—namely, their three-dimensional shape—is processed by both inferotemporal cortex (Janssen et al., 2000a,b) and area AIP (Srivastava et al., 2009; Verhoef et al., 2010). However, IT neurons' activity start shortly after the visual presentation while area AIP becomes active later on, leading some authors to suggest that the former plays a role in the formation of a perceptual decision and in the monkey behavioral choice; while the latter would reflect the three-dimensional features of the stimulus only after perceptual decision formation (Verhoef et al., 2010).

All the studies so far reviewed converge in indicating that (1) two cortical areas (IT and AIP) are involved in the parallel processing of the same information on objects (size, shape, etc), (2) they share some neuronal properties, and (3) are tightly interconnected one with the other. However, while part of the posterior parietal cortex, in particular area AIP, is devoted to extract object affordances for pragmatic purposes, the inferotemporal areas encode object features for object recognition. This latter conclusion somehow reminds a categorical, anatomo-functional distinction between perceptual and pragmatic functions of the “visual brain in action” (Milner and Goodale, 1993). However, it might be suggested that “*objects, as pictorially described by visual areas, are devoid of meaning. They gain meaning because of an association between their pictorial description (meaningless) and motor behavior (meaningful)”* (Rizzolatti and Gallese, 1997). Thus, in this view, although pragmatic and pictorial aspects of object processing might play partially distinct roles in mediating behavior within specific contexts, they would jointly contribute to our qualitative, phenomenological perceptual experience of the outside world. An interesting fMRI experiment on human subjects provides direct support to this claim. Grefkes et al. (2002) asked human volunteers to recognize whether an object was identical to another one previously assessed by the same subject. Objects were abstract three-dimensional solids differing one from the other only in size and shape (not weight, texture, etc.), and the two objects could be assessed and recognized either visually or by tactile manipulation. The results showed that human area AIP was specifically activated when cross-modal matching of visual and tactile object features was required, even when no specific motor act had to be performed on the perceived object, thus supporting the role of this area in the processing of multimodal information about object shape.

Noteworthy, the possible link between pragmatic and semantic cross-modal processing of object features is even more evident if one considers the network of areas connected with area AIP. On one side, AIP has reciprocal connections with a sector of the secondary somatosensory cortex (Disbrow et al., 2003; Borra et al., 2008) which is particularly active during haptic exploration of objects (Krubitzer et al., 1995; Fitzgerald et al., 2004) and tactile object recognition (Reed et al., 2004). On the other, as already mentioned, AIP is connected with inferotemporal areas of the middle temporal gyrus, which convey semantic information on object identity (Borra et al., 2008). Thus, it is not surprising that cortical lesions involving AIP not only impair visually guided grasping (Gallese et al., 1994; Tunik et al., 2005), but also cause deficits in active tactile shape recognition, in the absence of (Valenza et al., 2001) or in association with (Reed and Caselli, 1994) tactile agnosia.

Taken together, all these data strongly indicate that AIP plays a crucial role in visuomotor transformation for visually- and somatosensory-guided manipulation of objects, but both pragmatic and pictorial information are involved in this process, likely contributing not only to the efficient organization of hand actions, but also to our phenomenological perceptual experience of objects.

## Space-dependent coding of objects affording self or others' action

The studies so far reviewed demonstrate that seeing an object, such as an apple, simultaneously activates parallel neuronal representations of its pictorial features and motor affordances, providing a comprehensive perceptual experience of the object itself. However, several recent studies evidenced that affordances can be modulated by different contextual factors (Costantini et al., 2010, 2011a,b; Borghi et al., 2012; Ambrosini and Costantini, 2013; Kalenine et al., 2013; Van Elk et al., 2014) and, among these latter, one of the most crucial is represented by the space in which objects are located. Is an apple processed and perceived in the same way when it is at hand, on the table in front of me, as when it is out of reach, on the top of the apple tree?

According to Poincaré (1908), “it is in reference to our own body that we locate exterior objects, and the only special relations of these objects that we can picture to ourselves are their relations with our body.” A similar idea has been expressed more recently by Gibson (1979), according to whom the abstract concept of space is only a conceptual achievement, while the perception of space is intimately linked with the guidance of our behavior in a crowded and cluttered environment. Thus, our capacity to act with our own body on the external world appears to be, theoretically, of crucial importance in establishing the way our brain process information on objects.

Although some previous behavioral studies in humans suggested that object affordances might not be influenced by the location in space of the observed object (Tucker and Ellis, 2001), recent behavioral (Costantini et al., 2010, 2011a; Ambrosini and Costantini, 2013) and TMS (Cardellicchio et al., 2013) studies suggest that the extraction of affordances and the recruitment of motor representations of graspable objects crucially depend on whether the object falls within the peripersonal, reachable space of the observer, in line with the classical philosophical and psychological models described above. While affordance effects are typically studied in relation to potential motor acts allowing one to approach and interact with an object, Anelli et al. (2013) demonstrated that potentially noxious objects (e.g., cactus, scorpio, broken bulb, etc.) induce an aversive affordance, which triggers in the observer's motor system the representation of escaping-avoidance reactions, particularly when the dangerous stimulus moves toward the observer's peripersonal space. Taken together, these findings support the idea that object processing is strictly related with the object spatial location, and that the peripersonal space is the most relevant source of information for affordance extraction.

According with the aforementioned concept of space, one would expect that the link between object affordances and the observer's peripersonal space relies on a pragmatic, rather than metric, reference frame. In other terms: is the physical distance of the object from the observer the crucial variable to gate affordance effect (metric representation) or does it depend on the observer's possibility to directly interact with the object (pragmatic representation)? The study by Costantini et al. (2010) addressed this issue by means of a behavioral paradigm exploiting the spatial alignment effect. In this study, subjects were visually presented with an object which could be located within or outside their peripersonal space, and the results evidenced the presence of an object affordance effect only when the object was located in the observer's peripersonal space. Crucially, if a transparent barrier was interposed between the subject and the object, although this latter was within the observer's peripersonal space (same metric distance), the affordance effect vanished as if the object were located in the extrapersonal space. Thus, the power of an object to automatically evoke potential motor acts appears to be strictly linked to the effective possibility of the onlooker to interact with it. Based on these findings, one would expect that seeing an object out-of-reach does not induce any activation of the observer's motor system, thus object perception should completely rely on posterior visual areas. In another behavioral study, Costantini et al. (2011b) replicated the finding that the affordance effect is evoked only when the object falls within the observer's peripersonal space, not when it is located in the extrapersonal space. However, they added a further interesting condition in which another individual (a virtual avatar) was sat close to the object presented in the extrapersonal space (see also Creem-Regehr et al., 2013): in this condition, the affordance effect was restored, showing that objects can afford suitable motor acts to interact with them when they are ready not only for the subject's hand, but also for another agent's hand. In line with this view, recent monkey (Ishida et al., 2010) and human (Brozzoli et al., 2013, 2014) studies showed that neuronal populations do exist in parietal and ventral premotor cortex encoding the spatial position of objects relative to both one's own body and the corresponding body part of an observed subject, suggesting the existence of a shared representation of the space near oneself and others.

### Canonical and canonical-mirror neurons: motor representations of objects and actions in space

The behavioral evidence so far reviewed suggest that the peripersonal space and social contexts in which an object is seen play a crucial role in affecting the likelihood that it will trigger potential motor representations in the observer's brain. However, the cortical mechanisms and neural bases underlying these processes need to be further investigated.

**Figure 1:**
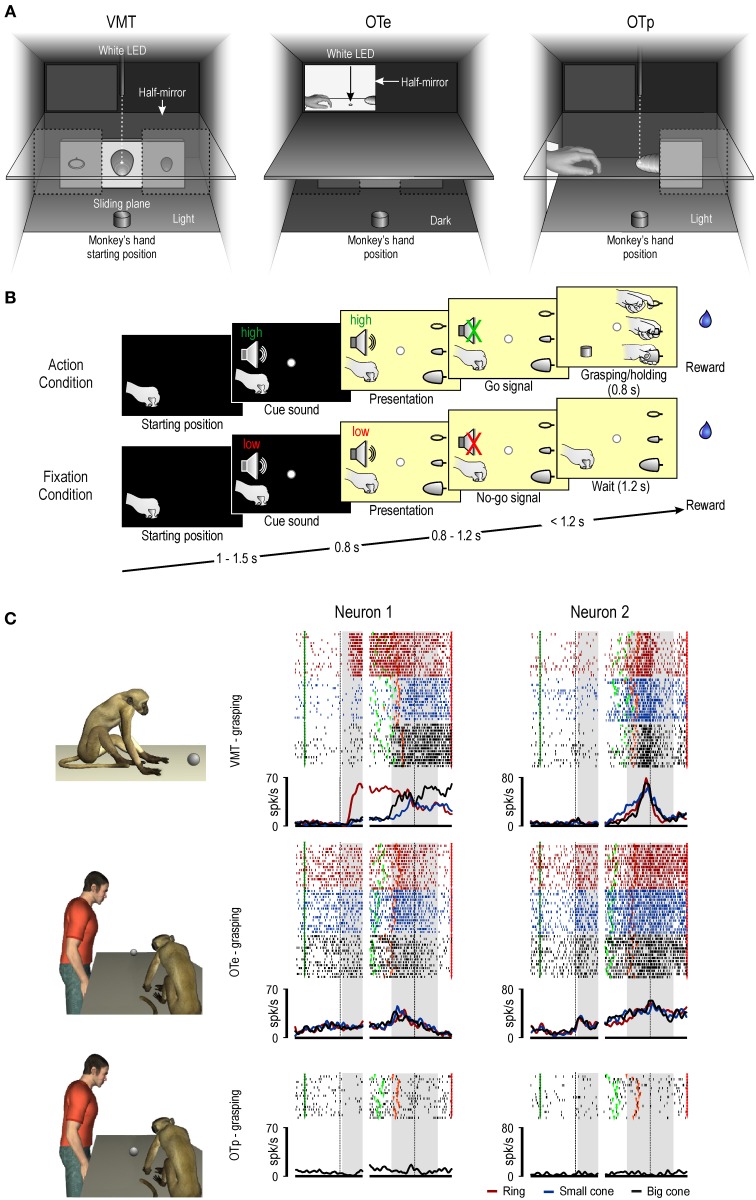
**(A)** Box and apparatus (seen from the monkey's point of view) settled for carrying out the visuomotor task (VMT), the observation task in the monkey's extrapersonal (OTe) and peripersonal (OTp) space. **(B)** Task phases of Action and Fixation conditions. Each trial started when the monkey had its hand in the starting position. A fixation point was presented and the monkey was required to fixate it for the entire duration of the trial. One of two cue sounds was then presented: a high tone, associated with the action trials, and a low tone, associated with fixation trials. After 0.8 s the lower sector of the box was illuminated and one of the three objects became visible. Then, after a variable time lag (0.8–1.2 s), the sound ceased (go/no-go signal) and the monkey either reached, grasped, and pulled the object (Action condition) or remained still for 1.2 s (Fixation condition) in order to receive the reward. The sequence of events and temporal constraints of the OTe and OTp were the same as in the monkey VMT, and the monkey had to simply maintain fixation in order to get the reward. **(C)** Examples of canonical-mirror neurons recorded in all the task contexts. On the left, a schematic view of the experimental paradigm. Each panel shows, from top to bottom, rastergrams and the spike density function. The gap in the rastergrams and histograms is used to indicate that the activity on its left side has been aligned on object presentation (first dashed black vertical line) while that on its right side is aligned on the pulling onset (second dashed black vertical line) of the same trial. The gray shaded areas indicate the time windows used for statistical analysis of neuronal response to object presentation (on the left) and grasping (on the right). Markers: dark green, cue sound onset; light green, cue sound offset (go signal); orange, detachment of the hand from the starting position (reaching onset); red, reward delivery at the end of the trial.

Before discussing recent data on these issues, it must be remembered that area F5 contains two main categories of visuomotor neurons, namely, canonical and mirror neurons. The neurons of these two categories show the same response during movement execution, while they differ in the type of visual stimulus triggering them. Canonical neurons, as previously described, respond only when the monkey observes an object, whereas mirror neurons activate only during observation of a motor act performed by another individual. In a recent neurophysiological study (Bonini et al., 2014), we recorded the activity of canonical and mirror neurons from the hand field of macaque ventral premotor cortex while the monkey performed a visuomotor task or observed the same task done by an experimenter, either in the monkey's peripersonal or extrapersonal space (Figures 1A,B). One of the main findings of this study was that the previously proposed dichotomy between canonical and mirror neurons appears to be at least too rigid. Indeed, beyond the classical mirror and canonical neurons, grasping neurons have been found showing hallmark features of both categories, that is, they responded both to object presentation and to observation of other's action (“canonical-mirror” neurons—see Figure 1C).

A further important result of this study concerns the influence of the space sector in which a target object was presented on the response of these three categories of neurons. Mirror neurons could code others' action both when it was presented in the monkey's peripersonal and extrapersonal space, in line with previous studies (Caggiano et al., 2009). In contrast, object coding by canonical neurons appeared to be markedly constrained to the peripersonal space, as well as to the visual perspective (subjective view) from which the object was seen by the monkey. This is in line with the classical proposal maintaining that canonical neurons provide a representation of the potential motor act afforded by the observed object, likely participating in the visuomotor transformations of object properties into the appropriate motor act for grasping it (Jeannerod et al., 1995; Fogassi et al., 2001).

Canonical-mirror neurons evidenced different response patterns. Example Neuron 1 (Figure 1C) would be classified as a canonical neuron, based on the VMT, but it also responded during the observation of the other's action performed in the extrapersonal space. Example Neuron 2 (Figure 1C), in contrast, did not show any response to the presentation of the object during the VMT, while it responded both to objects presented in the monkey's extrapersonal space and the subsequent experimenter's action. This latter finding suggests that the response of part of the canonical-mirror neurons to object presentation should not play a relevant role in visuomotor transformations for grasping. Rather, the object-triggered activation of canonical-mirror neurons may provide a *predictive* representation of the impending action of the observed agent.

**Figure 2:**
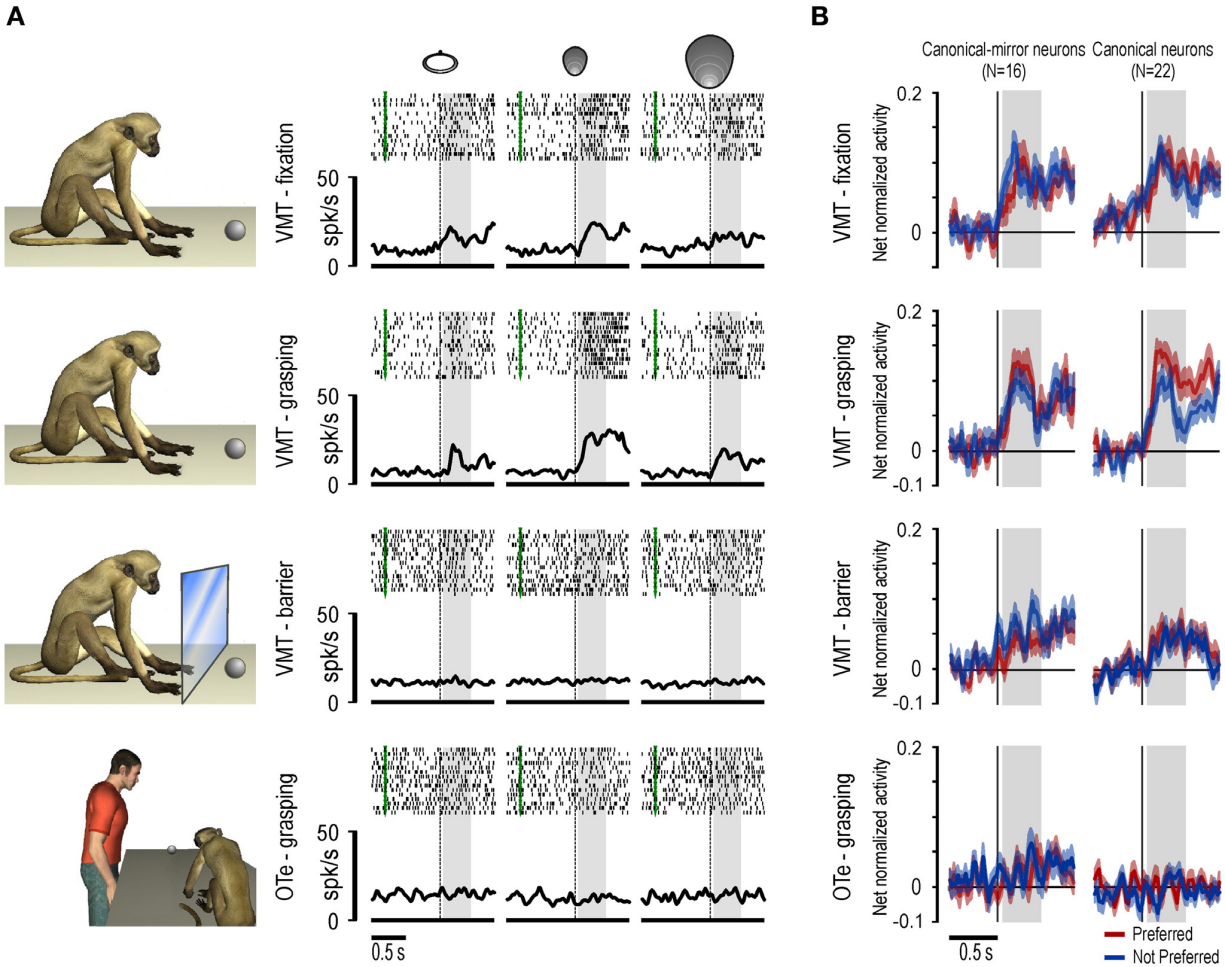
**(A)** Example of a canonical neuron recorded during an additional control experiment in which the object was presented behind a transparent plastic barrier. Note that the response during object presentation in the VMT was abolished with the interposition of the barrier. Only the alignment and the time window related to object presentation are shown. Other conventions as in Figure 1. **(B)** Time course and intensity of the population activity of canonical-mirror and canonical neurons relative to the preferred (red) and not preferred (blue) target object. For each neuron, the preferred/not preferred object are those triggering the stronger/wicker response during grasping execution. The activity is aligned on the light onset during different tasks and conditions.

In the same study we also showed that space-constrained coding of object, both by canonical and canonical-mirror neurons, relies on a pragmatic rather than metric representation of space. Indeed, most (about 75%) of the recorded canonical and canonical-mirror neurons discharged weakly to object presentation when it occurred behind a transparent plastic barrier, with about half of them showing no significant activation in this condition (see Figures 2A,B). This finding clearly demonstrates that neuronal responses to object rely on the actual possibility for the monkey to interact with the observed stimulus. This effect can be explained by the anatomical connections of this sector of area F5 with the adjacent area F4 (Matelli et al., 1986), whose neurons encode monkey's peripersonal space in a pragmatic format (Fogassi et al., 1996).

Space-constrained coding of objects as potential targets for self and others' action appears to rely on different types of neurons located in the same area: some of these neurons, which might enable motor prediction, can play a role for planning actions and for preparing behavioral reactions in the physical and social world.

## Conclusions

Most of the reviewed studies indicate that, besides the purely pictorial description of objects occurring in higher order visual areas, the processing of object features also involves different parallel parieto-frontal circuits constituting the extended motor system (Rizzolatti and Luppino, 2001). In these circuits affordances and contextual elements are crucial for a pragmatic object representation. Among them the peripersonal space appears to play a pivotal role in gating the representation of the potential motor act afforded by the object. When the object is located in the extrapersonal space, its representation as a potential target for the observer's hand action is not activated, while a motor representation of the object appears to be triggered if this latter is a potential target for an observed agent.

### Conflict of interest statement

The authors declare that the research was conducted in the absence of any commercial or financial relationships that could be construed as a potential conflict of interest.
